# Impacts of injury severity on long-term outcomes following motor vehicle crashes

**DOI:** 10.1186/s12889-021-10638-7

**Published:** 2021-03-27

**Authors:** Kevin K. C. Hung, Annette Kifley, Katherine Brown, Jagnoor Jagnoor, Ashley Craig, Belinda Gabbe, Sarah Derrett, Michael Dinh, Bamini Gopinath, Ian D. Cameron, On behalf of the FISH Investigators

**Affiliations:** 1 Accident and Emergency Medicine Academic Unit, Chinese University of Hong Kong, Hong Kong SAR, China; 2 John Walsh Centre for Rehabilitation Research, Sydney Medical School Northern, Level 13, Kolling building, St Leonards, Sydney, NSW Australia; 3 The George Institute for Global Health, Sydney, Australia; 4 School of Public Health and Preventive Medicine, Monash University, Melbourne, Australia; 5 Injury Prevention Research Unit (IPRU), Preventive & Social Medicine, Dunedin, New Zealand; 6 Royal Prince Alfred Hospital, The University of Sydney, Sydney, Australia; 7 Macquarie University Hearing, Department of Linguistics, Macquarie University, Sydney, NSW Australia

**Keywords:** Health related quality of life, Return to work, Road injuries, Recovery

## Abstract

**Background:**

There is growing evidence that a range of pre-injury, injury related and post-injury factors influence social and health outcomes across the injury severity spectrum. This paper documents health related outcomes for people with mild, moderate and severe injury after motor vehicle crash (MVC) injuries in New South Wales, Australia.

**Methods:**

This inception cohort study followed 2019 people injured in MVCs, for 6 and 12 months post-injury. We categorised moderate injury as hospital length-of-stay (LOS) of 2–6 days and Injury Severity Score (ISS) of 4–11, while severe injury as LOS ≥7 days or ISS ≥ 12. We examined differences in paid work status, 12-Item Short Form Survey (SF12), EQ-5D and World Health Organisation Disability Assessment Schedule II (WHODAS) outcomes longitudinally from baseline to 12 months between levels of injury severity using linear mixed models for repeated measures. We first considered minimally sufficient adjustment factors (age, sex, crash role, perceived danger in crash, pre-injury health, pre-injury EQ-5D, recruitment source), and then more extensive adjustments including post-injury factors. The presence of mediating pathways for SF-12 Physical Component Summary (PCS) and Mental Component Summary (MCS) via post-injury factors was evaluated.

**Results:**

Based on hospital length of stay (LOS), 25 and 10% of participants sustained moderate and severe injuries, respectively, while 43 and 4% had these injuries based on ISS. Twelve months post-injury LOS ≥7 days versus ≤1 day was associated with an estimated 9 units lower mean SF12 PCS using a minimally sufficient adjustment model, and LOS ≥ 7 days was associated with a 3 units lower mean SF12 MCS score. Mediation analyses (LOS ≥ 7 days vs ≤1 day) found for SF12 MCS outcomes, effects of injury severity were small and mostly indirect (direct effect − 0.03, indirect effect − 0.22). Whereas for SF12 PCS outcomes the effect of having a more severe injury rather than mild were both direct and indirect (direct effect − 0.50, indirect effect − 0.38).

**Conclusions:**

Individuals with severe injuries (those with LOS ≥ 7 days and ISS 12+) had poorer recovery 12 months after the injury. In addition, post-injury mediators have an important role in influencing long-term health outcomes.

**Trial registration:**

Australia New Zealand Clinical trial registry identification number - ACTRN12613000889752.

**Supplementary Information:**

The online version contains supplementary material available at 10.1186/s12889-021-10638-7.

## Background

Injuries accounted for 10% of the global burden of disease (GBD) in 2013, with road traffic injuries (RTI) being the main contributor to disability adjusted life years due to injury [1]. The World Health Organization Global Status Report on Road Safety 2018 highlighted the rising numbers of road traffic deaths reaching 1.35 million in 2016, and RTI were the leading cause of death among those aged 5–29 years worldwide [2]. In Australia, RTI accounted for 1132 injury deaths during 2014–15; 9% of all injury deaths during this period [3].

Injury severity measures for motor vehicle crashes (MVC) vary with the source of data from different jurisdictions [4]. Common data sources include police crash reports, hospital records, and insurance agencies. Due to the lack of established linkage with hospital data and the availability of timely access to other data sources, admission to hospital and the length of hospital stay are often used in police and transportation authority official reports [4]. Multiple studies have considered hospital length of stay (LOS) reliability compared to specific injury severity measures such as the Abbreviated Injury Scale (AIS) [5], Injury Severity Score (ISS) [6] and hospital resource use (major surgery, transfusion, or prolonged ventilation) [6], and found that it to be a reasonable proxy measure with moderate to high discriminatory value in identifying serious injury.

Previous studies have studied long term social and health outcomes after moderate to severe injuries (defined with either LOS, ISS or other means) [7–16]. A 2017 systematic review found severe injuries were generally associated with poorer quality of life, and particularly in the physical domain [7]. Berecki-Gisolf et al. found that the duration of work disability increased markedly with increasing LOS after MVC in Victoria, Australia [8]. At 17-months post injury, loss of earnings capacity was 35% among those with LOS ≥1 week (up to 67% in LOS ≥10 weeks), compared with 8% in those non-hospitalised [8]. Concerning HRQOL, Ameratunga et al. reported rate of 43% in hospitalised versus 20% in non-hospitalised crash drivers reported worse health (compared to pre-crash) at 18 months’ follow up [9]. After adjustment for age, sex, ethnicity, education and baseline health status, the effect was a 10-fold risk of worsened health among hospitalised crash drivers and a 3-fold risk among non-hospitalised drivers compared with a control group. It was also worth noting that further reductions in 36-Item Short Form Survey (SF36) domains scores of general and mental health, as well as vitality were seen despite improvements in physical function over time. The authors commented that this contradictory finding was puzzling, pointing toward potential unmet needs in these drivers which warranted further research.

Polinder et al. studied injured patients attending the emergency department (ED) in the Netherlands, and compared the 3 groups with non-hospitalised, LOS 1–3 days and ≥ 4 days and measured their health status EQ. 5D at 2.5, 5, 9 and 24 months [10]. They found that the health status EQ-5D was worse in those with longer hospital stay, and this pattern persisted at 24 months. Patients hospitalised for more than 3 days had limitations reported in mobility, performing usual activities and for pain and discomfort for 24 months [10]. Derrett et al. looked at disability measured by WHODAS among hospitalised and non-hospitalised injured patients, and found disability (defined by WHODAS of 10 or more) was more prevalent among the hospitalised at 3 months [11], but at 24 months the proportion was similar between hospitalised and non-hospitalised [12].

To date, few studies have recruited participants across a range of injury severity as measured by hospital LOS in MVC injured population. The Factors Influencing Social and Health outcomes after motor vehicle crash injury (FISH) study is an inception cohort study with participants recruited from mainly emergency departments, with 5% from other sources [17]. The inclusion of both metropolitan and rural Emergency Departments and hospital across New South Wales (NSW) allowed a more representative sample of the MVC injury population. The aim of this FISH paper is to describe and compare 6 and 12-month outcomes (work status, HRQOL, health status, disability and functioning) according to LOS and ISS [18].

## Method

The methods for the FISH have been previously described [17]. In summary, NSW residents aged 17 or older, injured in a MVC in NSW, Australia, between August 2013 and December 2016 were recruited from selected public hospital emergency departments (5% from other sources), within 1 month of the crash. Patients with pre-existing cognitive impairment e.g. dementia, injuries resulting from intentional self-harm and death of an immediate family member in the crash were excluded. Isolated, superficial soft tissue (very minor) injuries or extremely severe injuries defined by eligibility for the NSW Lifetime Care and Support Scheme including very severe traumatic brain injuries, spinal cord injuries, extensive burns or multiple amputations were also excluded from the study.

Eligible patients were contacted by telephone and a structured interview was conducted at baseline (within 28 days of the injury), 6 and 12 months following informed consent. The study was conducted according to the Declaration of Helsinki and approved by the Central Sydney (Concord Hospital) Local Health District Human Research Ethics Committee. Outcomes including return to work, HRQOL, health status, disability and functioning, psychological factors, pain and compensation were collected using validated tools at 6 and 12 months.

### Measures of injury severity

The baseline questionnaire asked participants if they had presented to the hospital due to MVC-related injury, and if so, the length of hospitalisation. Participants were divided into three groups according to self-reported hospital LOS (LOS ≤ 1 day – including those not presenting to hospital, LOS 2–6 days and LOS ≥ 7 days). These groups were based on the commonly defined cut-off for serious injuries according to the International Traffic Safety Data and Analysis Group [4].

We also used ISS to classify injury severity in the supplementary analyses. ISS is based on the anatomical injury severity classification of AIS [18]. The AIS classifies individual injuries by body region into 6 severity categories, with AIS 1 being minor and AIS 6 being maximal and untreatable [19]. ISS is calculated as the sum of the squares of the highest AIS code in each of the 3 most severely injured body regions of head or neck, face, thorax, abdomen, extremities, and external [18]. In the current study, we define ISS 1–3 as minor (with maximum AIS of 1), and ISS 12+ as severe (with at least 2 moderate or serious injuries).

ISS were derived using standard methods [18, 19] by a trained coder (KB). Data sources for the ISS coding were the AIS with all injuries specified for 51.3% of records, text data from the research data set for 34.7% of records, existing ISS data for 12.1% of records, a combination of ISS and AIS data for 0.4% of records where there were discrepancies between the two measures; an ISS of 1 was assigned to 0.4% of records with no injury information. ISS was directly calculated from the AIS in the first category above, in the second category the coder read all available text information and completed the standard calculation to derive the ISS. In the third category, the pre-existing ISS and AIS data were inconsistent, so all available data was used to assign the lowest feasible ISS.

All coding was completed in accordance with the AIS coding guideline of coding conservatively, that is, when multiple AIS codes could apply to the available data, assign the least severe AIS code in that injury category” [19].

### Baseline variables, social and health outcomes collected

The baseline data included sociodemographic characteristics, employment, pre-injury health (BMI and history of chronic disease), HRQOL, health status, lifestyle habits, pain, disability and functioning, psychological factors, health care utilisation, injury, crash related factors, work and social life (social satisfaction and participation). During 6 and 12-months follow up, work, social life, health status and HRQOL, compensation, disability and functioning, psychological factors and pain were assessed. All psychometric scales used have been shown to be valid and reliable measures. The Short Form Survey (SF12) has 12 questions from the SF36 survey, and has two domains: the physical component summary (PCS) and the mental component summary (MCS) [20]. Higher PCS and MCS scores indicate better physical and mental wellbeing. The telephone administered version of EQ-5D-3L measures health status across five dimensions including mobility, self-care, usual activities, pain or discomfort, and anxiety or depression [21]. Each dimension has three response options (e.g. no, some and major problems), from which an overall summary index can be derived, based on health state valuations of each possible health state where 1 represents full health, 0 represents dead, and negative values represent health states valued as worse than dead and have a lower bound of − 1 [22].

Orebro Musculoskeletal Pain Screening Questionnaire (OMPSQ, short form) is a screening tool that predicts failure to return to work following a soft tissue injury and includes 10 items with the total score ranging between 1 (lowest risk) to 100 (highest risk), with a score of > 50 indicating a higher risk for future work disability [23]. The World Health Organization Disability Assessment Schedule II 12-item version (WHODAS II) has six domains including cognition, mobility, self-care, getting along, life activities and participation, ranging from no disability (0) to full disability (100) [24]. The Depression, Anxiety and Stress Scale (DASS-21) is a 21-item scale that provides a general assessment of psychological distress, depressive mood, anxiety and stress [25]. Impact of Events Scale – Revised (IES-R) is based on 22 self-reported items assessing subjective distress following traumatic events [26]. Further details of the study protocol have been published in the protocol paper [17].

### Statistical analyses

Two sets of analyses were developed. Our primary analyses examined the associations of hospital length of stay with the outcomes of disability and functioning (WHODAS II), health status and HRQOL (EQ-5D summary score and SF-12) and work status. Supplementary analyses examined the association of ISS with these outcomes. Hospital length of stay was used for the primary analysis because it was collected in a consistent manner for all FISH participants based on participant self-report. Statistical analyses were performed using SAS v 9.4 (SAS Institute Inc., Cary, NC), MPLUS Version 7.3 and DAGGITY statistical software.

First, we examined descriptive statistics on baseline characteristics by subgroups of injury severity using means (SD), frequencies and percentages with chi-square tests, t-tests and general linear model F tests (Step 1). Second, we examined descriptive statistics for a variety of longitudinal health-related and psychological outcomes from baseline to 12 months, again using means (SD), frequencies and percentages with chi-square tests, t-tests and general linear model F tests (Step 2).

Third, we evaluated differences in paid work, SF12, EQ-5D summary score and WHODAS outcomes longitudinally from baseline to 12 months between levels of injury severity after adjusting for relevant covariates, using linear mixed models for repeated measures with unstructured serial correlation between time points within individuals. Consideration was given to the roles of the following factors via directed acyclic graphs: age, sex, preinjury health (comorbidities), preinjury EQ. 5D, education, preinjury work, recruitment source, social satisfaction, preinjury history of anxiety or depression, crash role, perceived danger in crash, hospital admission (for models of ISS level, not in models of hospital stay as a proxy measure for injury severity), pain at baseline, pain catastrophising (pain catastrophising scale) at baseline, DASS-21 and IES-R scores at baseline, compulsory third party insurance (CTP) claimant status. DAGGITY software was used to construct and examine the directed acyclic graphs, while SAS was used to run the linear mixed models. Adjusted model results (beta coefficients, 95% confidence intervals and *p* values) are presented for two models. Model 1 adjusted for minimally sufficient adjustment factors for both exposure and outcome: age, sex, crash role, perceived danger in crash, preinjury health, preinjury EQ. 5D, and recruitment source. Model 2 adjusted for all factors hypothesised to underlie either exposure or outcome status, including post-injury factors. Additionally, these models were adjusted for education, preinjury work, social satisfaction, preinjury history of anxiety or depression, pain at baseline, pain catastrophising at baseline, DASS-21 and IES-R scores at baseline, and CTP claimant status. (Step 3).

Fourth, since post-injury factors including baseline SF12 scores, baseline pain, baseline psychological status and CTP claimant status are possible mediators of the impact of more severe injury on long-term outcomes, we evaluated the presence of these mediating pathways for SF-12 PCS and MCS using MPLUS Version 7.3 (Step 4). The direct effect is defined as the effect of exposure on the outcome without the mediator, whereas the indirect effect is when the effect of exposure works through the mediator on the outcome.

## Results

The FISH study recruited a total of 2019 participants. Of the 2019 participants recruited at baseline, the overall follow up rate was 73.5% for 6 months and 59.5% for 12 months. The follow up rate at 12-month was slightly lower among the group with LOS ≥ 7 days with 55.1%. Of 2018 participants who self-reported LOS, 1304 (64.6%) had stays of ≤1 day, 507 (25.1%) had 2–6 day stays, and 207 (10.3%) reported hospital LOS ≥ 7 days. For the supplementary analyses based on ISS, moderate injury (ISS 4–11) was present in 43% of participants while severe injury (ISS ≥12) was present in 4% of participants.

### Baseline socioeconomic characteristics

Participants in the FISH study were predominantly male (65%) and Australian-born (71%), ranging in age from 17 to 92 years (mean 41 years). The cohort includes motor vehicle occupants (46%), motorcyclists (31%), cyclists (14%), pedestrians and others (8%), recruited primarily from hospital emergency department presentations (95%) at a diverse range of metropolitan and regional locations.

Table 1 shows participants who reported hospital length of stay≥7 days were more likely to be older (mean 45.5 years), male (72%), born in Australia (78%), with English as their primary language (91%), recruited from regional hospitals (29%), and were less likely to have tertiary education (25%) or be in paid work or self-employment pre-injury (69%) compared to LOS ≤ 1 day (all *p* < 0.05).

**Table 1:** Baseline demographic, health, crash and injury-related characteristics by length of hospital stay

		Length of hospital stay		
	<=1 day (***n*** = 1304)	2–6 days (***n*** = 507)	7 days or more (***n*** = 207)	
	*Mean (SD) or* *n (%)*	*Mean (SD) or* *n (%)*	*Mean (SD) or* *n (%)*	*P value*
**Self-reported days in hospital (mean, SD)**	0.24 (0.43)	3.5 (1.3)	12.9 (6.6)	NA
**Age (mean, SD)**	39.7 (15.6)	43.1 (17.3)	45.5 (18.2)	< 0.0001
**Male gender**	784 (60.1)	372 (73.4)	149 (72.0)	< 0.0001
**Country of birth**				0.008
Australia	899 (68.9)	374 (73.8)	161 (77.8)	
New Zealand	35 (2.7)	16 (3.2)	7 (3.4)	
United Kingdom	79 (6.1)	38 (7.5)	10 (4.8)	
Other	291 (22.3)	79 (15.6)	29 (14.0)	
**English as primary language**	1171 (89.8)	478 (94.3)	188 (90.8)	0.01
**Marital status**				0.2
Divorced, widowed or separated	127 (9.8)	50 (9.9)	27 (13.0)	
Married or de facto	640 (49.1)	269 (53.2)	104 (50.2)	
Never married	536 (41.1)	187 (37.0)	76 (36.7)	
**Recruitment source**				< 0.0001
RNSH or RPAH	778 (59.7)	227 (44.8)	67 (32.4)	
Orange, Dubbo or Bathurst hospital	103 (7.9)	132 (26.0)	59 (28.5)	
Other hospital	338 (25.9)	136 (26.8)	74 (35.8)	
Non-hospital	85 (6.5)	12 (2.4)	7 (3.4)	
**Educational level**				< 0.0001
Primary or pre-primary	70 (5.4)	38 (7.5)	18 (8.7)	
Secondary	375 (28.8)	167 (32.9)	72 (34.8)	
Technical or other further education	302 (23.2)	121 (23.9)	65 (31.4)	
Tertiary or university	556 (42.7)	181 (35.7)	52 (25.1)	
**Pre-injury paid work or self-employment**	1011 (77.5)	378 (74.6)	143 (69.1)	0.02
**Pre-injury income (Aust dollars)**				0.5
$0–20,799	53 (5.5)	17 (4.8)	10 (7.4)	
$20,800–41,599	151 (15.7)	52 (14.7)	28 (20.7)	
$41,600–64,999	263 (27.3)	94 (26.5)	40 (29.6)	
$65,000–103,999	277 (28.8)	105 (29.6)	30 (22.2)	
$104,000+	219 (22.7)	87 (24.5)	27 (20.0)	
**BMI (mean, SD)**	26.1 (5.3)	26.6 (5.4)	26.9 (6.3)	0.1
**Any pre-injury comorbidity on list of 18 specific items**	726 (55.7)	286 (56.4)	128 (61.8)	0.2
**Current smoking**	222 (17.0)	87 (17.2)	40 (19.3)	0.7
**Alcohol intake - audit-C score (mean, SD)**	3.25 (2.56)	3.37 (2.54)	3.21 (2.86)	0.6
**Crash type**				< 0.0001
Car driver	522 (40.1)	143 (28.2)	58 (28.2)	
Car passenger	143 (11.0)	47 (9.3)	13 (6.3)	
Motorbike driver or passenger	329 (25.3)	197 (38.9)	102 (49.5)	
Bicyclist	210 (16.1)	76 (15.0)	13 (6.3)	
Pedestrian or skateboard	99 (7.6)	44 (8.7)	20 (9.7)	
**Perceived danger of death**				< 0.0001
Overwhelming	120 (9.3)	59 (11.9)	28 (14.3)	
Great	182 (14.1)	87 (17.5)	43 (21.9)	
Moderate	238 (18.5)	101 (20.4)	52 (26.5)	
Small	265 (20.6)	96 (19.4)	28 (14.3)	
None	482 (37.5)	153 (30.9)	45 (23.0)	
**Perceived danger of disability**				< 0.0001
Overwhelming	102 (8.6)	62 (13.5)	34 (18.1)	
Great	195 (16.5)	99 (21.6)	44 (23.4)	
Moderate	303 (25.6)	111 (24.2)	49 (26.1)	
Small	293 (24.8)	94 (20.5)	32 (17.0)	
None	291 (24.6)	93 (20.3)	29 (15.4)	
**Self-report of psychological injury in accident**	317 (24.3)	104 (20.5)	49 (24.7)	0.2
**Self-report of regions injured**				
Head or face	390 (29.9)	149 (29.4)	64 (30.9)	0.9
Neck	508 (39.0)	119 (23.5)	39 (18.8)	< 0.0001
Spine or back	520 (39.9)	175 (34.5)	82 (39.6)	0.1
Torso	505 (38.7)	271 (53.5)	124 (59.9)	< 0.0001
Upper extremity	893 (68.5)	351 (69.2)	122 (58.9)	0.017
Lower extremity	707 (54.2)	306 (60.4)	144 (69.6)	< 0.0001
**Self-report of predominant injury**				< 0.0001
Multiple areas	151 (13.0)	46 (9.9)	19 (9.7)	
Head/face	104 (9.0)	28 (6.0)	9 (4.6)	
Neck	143 (12.3)	19 (4.1)	9 (4.6)	
Spine/back	74 (6.4)	29 (6.3)	17 (8.7)	
Torso	212 (18.3)	123 (26.5)	56 (28.6)	
Lower extremity	198 (17.1)	115 (24.8)	68 (34.7)	
Upper extremity	278 (24.0)	104 (22.4)	18 (9.2)	

### Injury characteristics

Compared to individuals with hospital length of stay ≤1 day, those with stays ≥7 days were less likely to be car occupants (35%) and more likely to be motorcyclists (50%), have injuries involving the lower extremity (70%) or torso (60%) and perceive a substantial danger of death and disability in the accident (all *p* < 0.05) (Table 1).

### Health and psychological outcomes by length of initial hospital stay

In unadjusted analyses (Table 2), increasing injury severity as indicated by length of hospital stay was significantly associated with lower work participation, more reported pain, worse health related quality of life, more disability, more post-traumatic symptoms, and worse psychological states at 6 and 12 months (all *p* < 0.05). It was not clearly associated with pain catastrophising.

**Table 2:** Descriptive statistics on paid work (or self-employment), quality of life, disability, pain and psychological outcome measures by categories for length of hospital stay

	Pre-Injury	Baseline post-injury	6 months	12 months
	Mean (SD) or N (%)	Mean (SD) or N (%)	Mean (SD) or N (%)	Mean (SD) or N (%)
	N (%)	N (%)	N (%)	N (%)
**Paid work**				
LOS < = 1 day	1011 (77.5)		Incomplete	581 (76.8)
LOS 2–6 days	378 (74.6)		Incomplete	234 (71.1)
LOS ≥ 7 days	143 (69.1)		Incomplete	71 (64.0)
*p* value	0.02			0.006
*Among those in paid work pre-injury*				
LOS < = 1 day			665 (90.6)	536 (91.3)
LOS 2–6 days			245 (88.1)	216 (87.5)
LOS ≥ 7 days			70 (70.7)	67 (81.7)
*p* value			< 0.0001	0.015
**Full score (1) achieved on the total EQ-5D-3L summary score**				
LOS < = 1 day	878 (67.4)	73 (5.6)	416 (43.4)	387 (51.1)
LOS 2–6 days	357 (70.7)	4 (0.8)	106 (27.6)	133 (40.6)
LOS ≥ 7 days	143 (69.1)	0 (0)	19 (13.7)	17 (14.9)
*p* value	0.3	< 0.0001	< 0.0001	< 0.0001
**Any pain**				
LOS < = 1 day	Not available	1087 (83.4)	564 (60.3)	388 (51.2)
LOS 2–6 days	Not available	468 (92.3)	281 (74.1)	201 (61.1)
LOS ≥ 7 days	Not available	199 (96.1)	126 (90.0)	97 (85.1)
*p* value		< 0.0001	< 0.0001	< 0.0001
	**Mean (SD)**	**Mean (SD)**	**Mean (SD)**	**Mean (SD)**
**EQ-5D-3L summary score**				
LOS < = 1 day	0.93 (0.14)	0.49 (0.35)	0.81 (0.25)	0.85 (0.23)
LOS 2–6 days	0.93 (0.14)	0.29 (0.36)	0.74 (0.27)	0.78 (0.27)
LOS ≥ 7 days	0.92 (0.14)	0.09 (0.37)	0.61 (0.30)	0.67 (0.26)
*p* value	0.7	< 0.0001	< 0.0001	< 0.0001
**SF12-PCS**				
LOS < = 1 day	Not available	38.0 (11.2)	48.6 (9.8)	49.9 (9.3)
LOS 2–6 days	Not available	29.3 (9.2)	44.9 (10.7)	47.0 (11.0)
LOS ≥ 7 days	Not available	25.1 (8.4)	37.8 (11.4)	39.6 (11.5)
*p* value		< 0.0001	< 0.0001	< 0.0001
**SF12-MCS**				
LOS < = 1 day	Not available	49.2 (12.0)	51.9 (10.5)	52.9 (9.4)
LOS 2–6 days	Not available	48.9 (11.7)	52.4 (10.5)	52.1 (10.9)
LOS ≥ 7 days	Not available	46.7 (12.1)	48.9 (13.1)	50.2 (11.4)
*p* value		0.02	0.003	0.02
**WHODAS**				
LOS < = 1 day	Not available	Not available	9.9 (16.3)	8.3 (15.9)
LOS 2–6 days	Not available	Not available	13.5 (18.7)	12.0 (18.3)
LOS ≥ 7 days	Not available	Not available	23.7 (22.7)	23.0 (22.1)
*p* value			< 0.0001	< 0.0001
**IES-R total**				
LOS ≤ 1 day	Not available	3.53 (3.12)	2.18 (2.75)	1.66 (2.54)
LOS 2–6 days	Not available	3.75 (3.10)	2.49 (2.98)	2.17 (2.79)
LOS ≥ 7 days	Not available	4.03 (3.16)	3.57 (3.27)	3.59 (3.33)
*p* value		0.06	< 0.0001	< 0.0001
**DASS-21 total**				
LOS ≤1 day	Not available	12.3 (15.1)	9.6 (14.3)	7.3 (12.7)
LOS 2–6 days	Not available	13.1 (15.1)	10.1 (14.4)	8.7 (13.0)
LOS ≥ 7 days	Not available	14.7 (15.4)	15.1 (16.8)	14.7 (16.1)
*p* value		0.09	0.0002	< 0.0001
**Perceived change / progress (−5 to + 5)**				
LOS ≤ 1 day			3.18 (2.33)	3.58 (2.06)
LOS 2–6 days			2.64 (2.30)	3.14 (2.17)
LOS ≥ 7 days			1.96 (2.49)	2.22 (2.33)
*p* value			< 0.0001	< 0.0001
**Numeric pain scale among those reporting any pain at each interview**^a^				
LOS ≤ 1 day		4.6 (2.3)	3.5 (2.3)	3.3 (2.2)
LOS 2–6 days		5.2 (2.2)	3.7 (2.4)	3.6 (2.3)
LOS ≥ 7 days		5.7 (2.2)	4.0 (2.2)	4.1 (2.2)
*p* value		< 0.0001	0.06	0.003
**Pain catastrophizing scale among those reporting any pain at each interview**^a^				
LOS ≤ 1 day		15.4 (13.6)	14.4 (13.9)	13.0 (13.6)
LOS 2–6 days		16.0 (14.1)	13.9 (14.6)	13.2 (13.8)
LOS ≥ 7 days		19.0 (14.0)	15.1 (13.7)	16.2 (15.3)
*p* value		0.003	0.7	0.12
**OMPSQ among those reporting current pain at each interview**				
LOS < = 1 day		39.6 (17.8)	36.0 (20.6)	34.1 (21.0)
LOS 2–6 days		45.4 (16.7)	38.5 (20.2)	38.2 (20.5)
LOS ≥ 7 days		51.8 (15.7)	46.0 (21.2)	46.4 (20.9)
*p* value		< 0.0001	< 0.0001	< 0.0001

^a^26 individuals who did not report pain at baseline missed out on the questions about pain and pain catastrophizing at their 6 month follow up interview

### Adjusted models of paid work, SF12, EQ. 5D and WHODAS by LOS

At 12 months post injury, after adjusting for a minimally sufficient set of adjustment factors (Model 1: age, sex, crash role, perceived danger in crash, preinjury health, preinjury EQ. 5D and recruitment source) in mixed models for repeated measures over time (Table 3), LOS > = 7 days versus <= 1 day was associated with an estimated 50% lower odds of being in current paid work, 9 units lower mean SF12 PCS, 0.15 units lower EQ. 5D summary scores, 13 units higher mean WHODAS disability scores, and 3 units lower mean SF12 MCS score (all *p* < 0.05). LOS 2–6 days versus <= 1 day was also associated, to a lesser degree, with lower SF12 PCS, SF12 MCS and EQ. 5D summary score and higher WHODAS disability scores (all *p* < 0.05).

**Table 3:** Adjusted mixed models for repeated measures over time

	Mean difference β (95% CI)	***p*** value	Mean difference β (95% CI)	***p*** value	Interaction ***p*** value*
	PRE INJURY		12 MONTH FOLLOW UP		
**PAID WORK**					
Model 1: Minimally sufficient adjustment at or before injury**					0.18
LOS ≤ 1 day	Ref		Ref		
LOS 2–6 days	−0.07 (−0.36, 0.22)	0.6	−0.23 (−0.59, 0.12)	0.19	
LOS ≥7 days	−0.25 (−0.66, 0.16)	0.2	−0.70 (−1.21, − 0.19)	0.007	
Model 2: Full adjustment including post-injury factors**					0.27
LOS ≤ 1 day	Ref		Ref		
LOS 2–6 days	−0.06 (− 0.37, 0.25)	0.7	− 0.22 (− 0.59, 0.15)	0.2	
LOS 7+ days	− 0.27 (− 0.69, 0.16)	0.2	− 0.67 (− 1.20,-0.14)	0.012	
	**BASELINE** **POST INJURY**		**12 MONTH** **FOLLOW UP**		
**SF12 PCS**					
Model 1: Minimally sufficient adjustment at or before injury**					< 0.0001
LOS ≤ 1 day	Ref		Ref		
LOS 2–6 days	−8.29 (−9.40, −7.18)	< 0.0001	−2.25 (−3.46, −1.04)	0.0003	
LOS ≥7 days	− 11.5 (−13.2, −9.8)	< 0.0001	−8.78 (− 10.7, −6.92)	< 0.0001	
Model 2: Full adjustment including post-injury factors**					< 0.0001
LOS ≤ 1 day	Ref		Ref		
LOS 2–6 days	−6.42 (−7.37, −5.46)	< 0.0001	−0.41 (−1.56, 0.74)	0.5	
LOS ≥7 days	− 8.49 (− 9.89, − 7.09)	< 0.0001	−5.64 (−7.41, −3.87)	< 0.0001	
**SF12 MCS**					
Model 1: Minimally sufficient adjustment at or before injury**					0.24
LOS ≤ 1 day	Ref		Ref		
LOS 2–6 days	−1.13 (−2.29, 0.03)	0.055	−1.65 (− 2.89, −0.40)	0.009	
LOS ≥7 days	−2.50 (−4.19, − 0.81)	0.0036	−3.05 (−4.96, − 1.13)	0.0018	
Model 2: Full adjustment including post-injury factors**					0.31
LOS ≤ 1 day	Ref		Ref		
LOS 2–6 days	0.18 (−0.73, 1.09)	0.69	−0.36 (− 1.48, 0.77)	0.53	
LOS ≥7 days	−0.51 (−1.83, 0.81)	0.44	−0.99 (− 2.73, 0.74)	0.26	
**EQ 5D summary score**					
Model 1: Minimally sufficient adjustment at or before injury**					< 0.0001
LOS ≤ 1 day	Ref		Ref		
LOS 2–6 days	−0.21 (− 0.25, − 0.17)	< 0.0001	− 0.06 (− 0.09, − 0.02)	0.0002	
LOS ≥7 days	−0.38 (− 0.43, − 0.32)	< 0.0001	−0.15 (− 0.20,-0.10)	< 0.0001	
Model 2: Full adjustment including post-injury factors**					< 0.0001
LOS ≤ 1 day	Ref		Ref		
LOS 2–6 days	− 0.16 (− 0.19, − 0.12)	< 0.0001	− 0.01 (− 0.04, 0.02)	0.53	
LOS ≥7 days	− 0.29 (− 0.34, − 0.24)	< 0.0001	− 0.07 (− 0.11,-0.02)	0.001	
**WHODAS**					
Model 1: Minimally sufficient adjustment at or before injury**					
LOS ≤ 1 day	–		Ref		
LOS 2–6 days	–		3.33 (1.13, 5.53)	0.003	
LOS ≥7 days	–		13.36 (9.96, 16.8)	< 0.0001	
Model 2: Full adjustment including post-injury factors**					
LOS ≤ 1 day	–		Ref		
LOS 2–6 days	–		0.73 (−1.18, 2.64)	0.45	
LOS ≥7 days	–		9.23 (6.27, 12.2)	< 0.0001	

Footnotes:

*Interaction *p* value for interaction between time point and ISS category

**Minimally sufficient adjustment factors for both exposure and outcome (Model 1): age, sex, crash role, perceived danger in crash, preinjury health, preinjury EQ. 5D, recruitment source

Full adjustment for all factors hypothesised to underlie either exposure or outcome status (Model 2), including post-injury factors: covariates from Model 1 PLUS education, preinjury work, social satisfaction, preinjury history of anxiety or depression, pain at baseline, pain catastrophising at baseline, DASS-21 and IESR scores at baseline, and CTP claimant status.

These findings were essentially the same on re-examining these models after adjusting for additional factors arising at or before the time of injury including educational level, preinjury work status, self-reported social satisfaction, and preinjury history of anxiety or depression (data not shown). If further adjusted for post-injury factors (Model 2: additional adjustment for baseline pain, baseline psychological status, CTP claimant status), LOS > = 7 days versus <= 1 day was still significantly associated with most 12 month outcomes except SF12 MCS (Table 3).

### Mediation analysis for SF12 outcomes at 12 months

The role of the post-injury factors (baseline SF12 scores, baseline pain, baseline psychological status and CTP claimant status) was explored using mediation analyses for SF12 PCS and MCS outcomes (Table 4). Baseline SF12 scores, baseline pain, baseline psychological status and CTP claimant status were all involved in indirect pathways of effect of injury severity on SF12 PCS and SF12 MCS at 12 months. For SF12 MCS outcomes, effects of injury severity were small and predominantly or entirely indirect, whereas for SF12 PCS outcomes the effect of having a more severe injury rather than a mild one were both direct and indirect.

**Table 4:** Mediation analysis for moderate-severe injury

	Total effect of LOS	Direct effect of LOS	Total of indirect effects of LOS^a^
	Standardised beta coefficient (SE) ^b^	Standardised beta coefficient (SE) [estimated % of total effect] ^b^	Standardised beta coefficient (SE) [estimated % of total effect] ^b^
**Outcome: SF12 PCS at baseline**			
LOS2–6 days vs 1 day or less	− 0.70 (0.05)	− 0.53 (0.04) [70%]	− 0.17 (0.03) [30%]
LOS ≥7 days vs 1 day or less	− 0.98 (0.07)	−0.74 (0.06) [75%]	− 0.24 (0.04) [25%]
**Outcome: SF12 PCS at 12 months**			
LOS2–6 days vs 1 day or less	− 0.24 (0.06)	0 (0.06) [Small or absent]	− 0.24 (0.03) [Predominantly or entirely indirect, estimated at 100%]
LOS > = 7 days vs 1 day or less	− 0.88 (0.09)	− 0.50 (0.09) [57%]	−0.38 (0.04) [43%]
**Outcome: SF12 MCS at baseline**			
LOS2–6 days vs 1 day or less	− 0.13 (0.05)	− 0.02 (0.04) [Small or absent]	−0.11 (0.03) [Predominantly or entirely indirect, estimated at 87%]
LOS > = 7 days vs 1 day or less	− 0.24 (0.07)	− 0.07 (0.06) [Small or absent]	−0.17 (0.04) [Predominantly or entirely indirect, estimated at 71%]
**Outcome: SF12 MCS at 12 months**			
LOS2–6 days vs 1 day or less	− 0.11 (0.06)	0.02 (0.06) [Small or absent]	− 0.12 (0.03) [Predominantly or entirely indirect, estimated at 91%]
LOS > = 7 days vs 1 day or less	− 0.25 (0.10)	− 0.03 (0.09) [Small or absent]	−0.22 (0.04) [Predominantly or entirely indirect, estimated at 87%]

^a^ Potential mediating factors for indirect effects of injury severity on SF12 included baseline pain, baseline psychological status and CTP claimant status. For SF12 outcomes at 12 months, the baseline value was also considered as a potential mediating factor

^b^ Estimated effects are standardised so that the beta coefficients describe standard deviations of SF12 PCS or SF12 MCS. Percentages for direct or indirect effects are shown when the confidence interval of the corresponding beta coefficient does not include 0

### Supplementary analyses based on categories of ISS

Analyses using ISS categories broadly supported overarching messages from the primary analysis and are available in Supplementary Materials. ISS and length of stay were strongly related (Table A1) with moderate Spearman correlation (ρ 0.43). Table A1 shows that the associations of sociodemographic and injury characteristics with injury severity as assessed by length of hospital stay are similar to those assessed using ISS category although there is a stronger association with alcohol intake.

Table A2 shows that more severe injury based on ISS category was associated with reduced work participation and poorer health status and HRQOL (EQ-5D, SF12 PCS, SF12 MCS) and WHODAS, particularly for ISS 9–11 and ISS 12 + .

Table A3 shows that these associations between return to paid work and health related outcomes over time by ISS category persist after minimally sufficient adjustment and full adjustment for factors hypothesised to underlie either exposure and outcome including post injury factors.

## Discussion

Participants with severe injury (as indicated by LOS ≥7 days) experienced distinct negative impacts on 12-month work participation, HRQOL, health status and disability. Baseline pain, baseline psychological status (DASS21, IESR, catastrophising), baseline SF12 and compensation claimant status were important predictors of long-term physical and mental health outcomes and were also mediators of the impact of injury severity on these health outcomes.

The negative impact of injury severity with long term outcome was consistent with previous findings [7–16], however the direct and indirect mediating effects have rarely been reported. With the ability to consider a comprehensive list of pre and post injury factors in our study, and the use of mediation analysis to look into direct and indirect effects of injury severity, this study provided new insights into the interplay of injury and other psychosocial factors affecting recovery.

In our study, we found effects of severe injury on 12 month outcomes (paid work, SF12 PCS, EQ. 5D and WHODAS) in both an extensive adjustment model (Model 2) and a minimally sufficient adjustment model (Model 1). Mediation analysis found 57% direct effect and 43% indirect effect for LOS (≥7 days vs 1 day or less) on SF12 PCS at 12 months, highlighting the role of mediating factors including the baseline pain, baseline psychological status (DASS21, IESR, catastrophising) and CTP claimant status. For SF12 MCS, mediation analysis showed predominantly or entirely indirect effect of injury severity on the 12-month outcome, so the effect of injury severity was seen only in the minimally sufficient adjustment model (Model 1).

Previous literature also supported pain, baseline psychological status and CTP claimant status as important factors for HRQOL and other outcomes [27–31]. Tournier et al. reported in whiplash injuries in the French and Spanish cohort, pain was found to be an intermediate factor between whiplash grading and overall health status and QOL [27]. In Norway, Tøien et al. compared trauma patients admitted to intensive care unit (ICU) with non-ICU trauma patients and the general population, and looked at the HRQOL 1 year after trauma [28]. It was found that depression at baseline (measured by hospital anxiety and depression scale HADS) was associated with lower SF36 scores in physical functioning, mental health, bodily pain and vitality domains at 12-months post injury, and higher IES score at baseline was associated with lower scores in general health and bodily pain domains [28]. Kenardy et al. studied CTP claimants in Queensland, Australian and found increasing pain level, PTSD, and major depressive episode lowered the PCS and MCS scores at 2 years [29]. It was surprising that minor injuries (measured by ISS 1–3) in this cohort reported the lowest MCS scores at 2 years, and a possible explanation was due to the limited representation of minor injuries and most of the patients had whiplash-related injuries (known association with poor recovery outcomes with chronicity of whiplash-related injuries).

Concerning the association of CTP claimant status with worse long-term outcomes, previous studies have suggested the possible mechanism influencing this [30, 31]. Littleton et al. reported lower SF36 PCS, greater anxiety (measured by HADS), and disability index (FRI) in patients claiming compensation after musculoskeletal injury after MVC [30]. Elbers et al. further explained this phenomenon by identifying factors that contributed to poor outcomes in the compensation process including the role of negative cognitions and the stress associated with the claims process [31]. Prior studies have also found early change in SF36 PCS and MCS score (from baseline to 1 year) was found to be important in predicting longer term outcomes [32], and therefore explaining the indirect mediation effect of baseline SF12 scores on the 12 month SF12 results.

Despite the strengths of this large, prospective cohort study, there are several limitations. The follow up rate was limited at 12 months especially for the hospital LOS ≥ 7 days (55.1%) and this may introduce responder bias for the 12-month results. To address this, we have used longitudinal mixed modelling and mediation analyses which take missing data into account based on an assumption that data are missing at random. Second, the proportion of the participants in the different hospital LOS groups are not equal in size, therefore some confidence intervals are quite wide especially for the groups with longer hospital LOS. Several authors have previously criticised the use of hospital LOS as an indicator for injury [5, 33–35], and among the criticisms included that the hospital LOS is not stable and is subjected to changes in delivery of healthcare [33, 35]. It was further demonstrated that the hospital LOS for those with serious long bone fractures has reduced considerably over the years since the 1980s [35]. However, hospitalisation and the length of stay is still considered the most common indicator used among transportation authorities worldwide [4]. Third, as with most self-reported outcome measures, the hospital length of stay and other outcomes in the questionnaires and follow up were subject to recall bias. However, the analyses using hospital length of stay and ISS are broadly similar. Lastly, we cannot rule out the possibility of residual confounding, such as the effect of alcohol [36, 37], despite our best attempts to collect and control for various factors including socioeconomic and injury related factors.

The implications for these findings are that clinicians should build on their current knowledge about people with more severe injuries being at risk of limited recovery. Post injury factors that can alert clinicians of the potential benefit of increased targeted treatment are the presence of severe pain, psychological distress and greater disability (meaning major limitations in self care and mobility).

## Conclusions

We found clear differences in recovery outcomes existing between the three hospital LOS groups in nearly all the outcomes at 6 and 12 months. Significantly less of those with LOS ≥ 7 days compared with LOS ≤ 1 day had paid work at 12 months after model adjustments. For physical health outcome (SF12 PCS) at 12 months, direct and indirect effect of injury severity both play a role, whereas for mental health outcome (SF-12 MCS) at 12 months, effects of injury severity were predominantly or entirely indirect. This finding has important implications demonstrating the role of mediators including baseline pain, baseline psychological status, CTP claimant status and baseline SF12 scores in influencing long term health outcomes in people with moderate to severe injury.

## Supplementary Information

**Additional file 1.**

## Data Availability

After the FISH study concludes data may be available from the corresponding author on specific request.

## Abbreviations

AIS: Abbreviated injury scale
CBT: Cognitive behavioural therapy
DASS-21: Depression, anxiety and stress scale
FISH study: Factors influencing social and health outcomes after motor vehicle crash injury study
GBD: Global burden of disease
HRQOL: Health-related quality of life
IES-R: Impact of events scale
ISS: Injury severity score
LOS: Length of stay
MVC: Motor vehicle crash
MCS: Mental component summary score
NSW: New South Wales
OMPSQ: Orebro musculoskeletal pain screening questionnaire
PCS: Physical component summary score
PTSD: Post traumatic stress disorder
SF12: 12-Item short form survey
WHODAS II: World health Organisation disability assessment schedule II
