# Supplementary material for: Impacts of injury severity on long-term outcomes following motor vehicle crashes
Source: BMC Public Health. 2021 Mar 27;21:602. doi: 10.1186/s12889-021-10638-7 (PMC8005247; doi:10.1186/s12889-021-10638-7)
Supplement: Supplementary file 1 — Additional file 1. [file 12889_2021_10638_MOESM1_ESM.docx]

Appendix: Injury Severity (as assessed by Injury Severity Score) and its associations

Table A1 Baseline demographic, health, crash and injury-related characteristics by categories of injury severity score (ISS)

|  |  |  | **ISS category** | |  |  |
| --- | --- | --- | --- | --- | --- | --- |
|  | **12+ (n=81)** | **9-11 (n=130)** | **4-8 (n=738)** | **1-3 (n=1070)** | |  |
|  | *Mean (SD) or  n (%)* | *Mean (SD) or  n (%)* | *Mean (SD) or  n (%)* | *Mean (SD) or  n (%)* | | *P value* |
| **Self-reported days in hospital (mean, SD) [interquartile range]** | 9.9 (8.1)  [5-15] | 6.3 (5.2)  [2-8] | 2.7 (2.4) [0-4] | 1.1 (0.9) [0-1] | | <0.0001 |
| **Age (mean, SD)** | 48.1 (14.5) | 45.9 (16.8) | 40.8 (15.9) | 40.3 (16.7) | | <0.0001 |
| **Length of stay** |  |  |  |  | | <0.0001 |
| ≤ 1 day | 7 (8.8) | 26 (20.0) | 395 (53.5) | 876 (81.9) | |  |
| 2-6 days | 27 (33.8) | 59 (45.4) | 262 (35.5) | 159 (14.9) | |  |
| 7+ days | 46 (57.5) | 45 (34.6) | 81 (11.0) | 35 (3.3) | |  |
| **Male gender** | 61 (75.3) | 90 (69.2) | 522 (70.7) | 632 (59.1) | | <0.0001 |
| **Country of birth** |  |  |  |  | | 0.08 |
| Australia | 63 (77.8) | 98 (75.4) | 538 (72.9) | 735 (68.7) | |  |
| New Zealand | 2 (2.5) | 4 (3.1) | 24 (3.3) | 28 (2.6) | |  |
| United Kingdom | 6 (7.4) | 8 (6.2) | 51 (6.9) | 62 (5.8) | |  |
| Other | 10 (12.4) | 20 (15.4) | 125 (16.9) | 245 (22.9) | |  |
| **English as primary language** | 77 (95.1) | 122 (93.9) | 678 (91.9) | 960 (89.7) | | 0.12 |
| **Marital status** |  |  |  |  | | 0.2 |
| Divorced, widowed or separated | 11 (13.6) | 20 (15.4) | 68 (9.2) | 105 (9.8) | |  |
| Married or defacto | 45 (55.6) | 63 (48.5) | 365 (49.5) | 541 (50.6) | |  |
| Never married | 25 (30.9) | 47 (36.2) | 304 (41.3) | 423 (39.6) | |  |
| **Recruitment source** |  |  |  |  | | <0.0001 |
| RNSH or RPAH | 29 (35.8) | 56 (43.1) | 392 (53.1) | 595 (55.6) | |  |
| Orange, Dubbo or Bathurst hospital | 17 (21.0) | 29 (22.3) | 162 (22.0) | 86 (8.0) | |  |
| Other hospital | 33 (40.7) | 40 (30.8) | 156 (21.1) | 320 (29.9) | |  |
| Non-hospital | 2 (2.5) | 5 (3.9) | 28 (3.8) | 69 (6.5) | |  |
| **Educational level** |  |  |  |  | | 0.002 |
| Primary or pre-primary | 3 (3.8) | 10 (7.7) | 52 (7.1) | 61 (5.7) | |  |
| Secondary | 19 (23.8) | 37 (28.5) | 239 (32.4) | 319 (29.8) | |  |
| Technical or other further education | 33 (41.3) | 39 (30.0) | 182 (24.7) | 234 (21.9) | |  |
| Tertiary or university | 25 (31.3) | 44 (33.9) | 265 (35.9) | 455 (42.6) | |  |
| **Pre-injury paid work or self-employment** | 61 (75.3) | 102 (78.5) | 575 (77.9) | 795 (74.3) | | 0.3 |
| **Pre-injury income** |  |  |  |  | | 0.8 |
| $0-20799 | 1 (1.8) | 6 (6.0) | 28 (5.1) | 45 (6.0) | |  |
| $20800-41599 | 7(12.3) | 12 (12.0) | 88 (16.2) | 124 (16.5) | |  |
| $41600-64999 | 14 (24.6) | 27 (27.0) | 146 (26.8) | 210 (28.0) | |  |
| $65000-103999 | 20 (35.1) | 35 (35.0) | 149 (27.3) | 208 (27.7) | |  |
| $104000+ | 15 (26.3) | 20 (20.0) | 134 (24.6) | 164 (21.8) | |  |
| **BMI (mean, SD)** | 26.8 (5.9) | 26.4 (5.1) | 26.3 (5.3) | 26.3 (5.6) | | 0.8 |
| **Any pre-injury comorbidity on list of 18 specific items** | 47 (58.0) | 67 (51.5) | 387 (52.4) | 639 (59.8) | | 0.012 |
|  | **12+ (n=81)** | **9-11 (n=130)** | **4-8 (n=738)** | **1-3 (n=1070)** | |  |
|  | *Mean (SD) or  n (%)* |  | *Mean (SD) or  n (%)* | *Mean (SD) or  n (%)* | | *P value* |
| **Current smoking** | 5 (6.2) | 24 (18.5) | 130 (17.6) | 190 (17.8) | | 0.06 |
| **Alcohol intake - audit-C score (mean, SD)** | 3.44 (2.37) | 3.11 (2.48) | 3.61 (2.59) | 3.05 (2.59) | | <0.0001 |
| **Crash type** |  |  |  |  | | <0.0001 |
| Car driver | 11 (13.6) | 33 (25.4) | 177 (24.0) | 502 (47.0) | |  |
| Car passenger | 12 (14.8) | 13 (10.0) | 47 (6.4) | 132 (12.4) | |  |
| Motorbike driver or passenger | 39 (48.2) | 50 (38.5) | 314 (42.6) | 225 (21.1) | |  |
| Bicyclist | 15 (18.5) | 17 (13.1) | 139 (18.8) | 128 (12.0) | |  |
| Pedestrian or skateboard | 4 (4.9) | 17 (13.1) | 61 (8.3) | 81 (7.6) | |  |
| **Perceived danger of death** |  |  |  |  | | 0.0005 |
| Overwhelming | 8 (10.8) | 19 (15.0) | 62 (8.6) | 118 (11.2) | |  |
| Great | 19 (25.7) | 29 (22.8) | 122 (16.8) | 143 (13.6) | |  |
| Moderate | 19 (25.7) | 31 (24.4) | 134 (18.5) | 207 (19.6) | |  |
| Small | 12 (16.2) | 17 (13.4) | 136 (18.8) | 224 (21.3) | |  |
| None | 16 (21.6) | 31 (24.4) | 271 (37.4) | 362 (34.4) | |  |
| **Perceived danger of disability** |  |  |  |  | | 0.016 |
| Overwhelming | 10 (14.1) | 20 (16.7) | 67 (10.0) | 102 (10.5) | |  |
| Great | 19 (26.8) | 35 (29.2) | 119 (17.7) | 165 (17.0) | |  |
| Moderate | 16 (22.5) | 26 (21.7) | 167 (24.9) | 254 (26.2) | |  |
| Small | 11 (15.5) | 21 (17.5) | 163 (24.3) | 224 (23.1) | |  |
| None | 15 (21.1) | 18 (15.0) | 156 (23.2) | 224 (23.1) | |  |
| **Self-report of psychological injury in accident** | 18 (22.2) | 38 (29.2) | 130 (17.6) | 285 (26.6) | | <0.0001 |
| **Self-report of regions injured** |  |  |  |  | |  |
| Head or face | 29 (35.8) | 43 (33.1) | 179 (24.3) | 352 (32.9) | | 0.0005 |
| Neck | 18 (22.2) | 26 (20.0) | 135 (18.3) | 488 (45.6) | | <0.0001 |
| Spine or back | 42 (51.9) | 46 (35.4) | 209 (28.3) | 480 (44.9) | | <0.0001 |
| Torso | 68 (84.0) | 78 (60.0) | 326 (44.2) | 429 (40.1) | | <0.0001 |
| Upper extremity | 64 (79.0) | 78 (60.0) | 516 (69.9) | 709 (66.3) | | 0.012 |
| Lower extremity | 45 (55.6) | 81 (62.3) | 453 (61.4) | 578 (54.0) | | 0.011 |
| **Self-report of predominant injury** |  |  |  |  | | <0.0001 |
| Multiple areas | 12 (15.8) | 12 (9.8) | 62 (9.2) | 130 (13.8) | |  |
| Head/face | 5 (6.6) | 5 (4.1) | 28 (4.1) | 103 (10.9) | |  |
| Neck | 0 (0) | 4 (3.3) | 21 (3.1) | 146 (15.5) | |  |
| Spine/back | 9 (11.8) | 10 (8.1) | 31 (4.6) | 70 (7.4) | |  |
| Torso | 32 (42.1) | 46 (37.4) | 181 (26.7) | 133 (14.1) | |  |
| Lower extremity | 10 (13.2) | 35 (28.5) | 165 (24.4) | 171 (18.1) | |  |
| Upper extremity | 8 (10.5) | 11 (8.9) | 189 (27.9) | 192 (20.3) | |  |

Table A2 Descriptive statistics on paid work (or self-employment), quality of life, disability, pain and psychological outcome measures categories of injury severity score (ISS)

|  | **Pre-Injury** | | **Baseline post-injury** | | **6 months** | | | **12 months** |
| --- | --- | --- | --- | --- | --- | --- | --- | --- |
|  | Mean (SD) or N (%) | | Mean (SD) or N (%) | | Mean (SD) or N (%) | | | Mean (SD) or N (%) |
|  | **N (%)** | | **N (%)** | | **N (%)** | | | **N (%)** |
| **Paid work** |  | |  | |  | | |  |
| ISS 12+ | 61 (75.3) | |  | | Incomplete | | | 31 (63.3) |
| 9-11 | 102 (78.5) | |  | | Incomplete | | | 59 (67.8) |
| 4-8 | 575 (77.9) | |  | | Incomplete | | | 332 (74.9) |
| 1-3 | 795 (74.3) | |  | | Incomplete | | | 464 (75.1) |
| p value | 0.3 | |  | |  | | | 0.15 |
| *Among those in paid work pre-injury* |  | |  | |  | | |  |
| ISS 12+ |  | |  | | 30 (69.8) | | | 31 (79.5) |
| 9-11 |  | |  | | 68 (82.9) | | | 57 (85.1) |
| 4-8 |  | |  | | 385 (89.1) | | | 307 (88.5) |
| 1-3 |  | |  | | 497 (89.7) | | | 424 (91.6) |
| p value |  | |  | | 0.0005 | | | 0.046 |
| **No problems at all on the EQ-5D-3L summary score** |  | |  | |  | | |  |
| ISS 12+ | 59 (72.8) | | 0 (0) | | 9 (15.5) | | | 10 (20.4) |
| 9-11 | 98 (75.4) | | 0 (0) | | 20 (19.6) | | | 24 (27.6) |
| 4-8 | 535 (72.6) | | 12 (1.6) | | 193 (34.3) | | | 200 (45.1) |
| 1-3 | 686 (64.3) | | 65 (6.1) | | 319 (42.1) | | | 303 (48.9) |
| p value | 0.0004 | | <0.0001 | | <0.0001 | | | <0.0001 |
| **Any pain** |  | |  | |  | | |  |
| ISS 12+ | Not available | | 79 (97.5) | | 50 (87.7) | | | 41 (83.7) |
| 9-11 | Not available | | 123 (94.6) | | 85 (84.2) | | | 59 (67.8) |
| 4-8 | Not available | | 674 (91.3) | | 384 (69.1) | | | 257 (57.8) |
| 1-3 | Not available | | 879 (82.2) | | 452 (61.0) | | | 329 (53.1) |
| p value |  | | <0.0001 | | <0.0001 | | | <0.0001 |
|  |  | |  | |  | | |  |
|  | **Mean (SD)** | | **Mean (SD)** | | **Mean (SD)** | | | **Mean (SD)** |
| **EQ-5D-3L summary score** |  | |  | |  | | |  |
| ISS 12+ | 0.94 (0.11) | | 0.12 (0.37) | | 0.61 (0.35) | | | 0.66 (0.30) |
| 9-11 | 0.93 (0.14) | | 0.20 (0.37) | | 0.69 (0.26) | | | 0.73 (0.27) |
| 4-8 | 0.94 (0.12) | | 0.33 (0.37) | | 0.78 (0.25) | | | 0.84 (0.20) |
| 1-3 | 0.91 (0.15) | | 0.49 (0.35) | | 0.82 (0.27) | | | 0.82 (0.27) |
| p value | 0.007 | | <0.0001 | | <0.0001 | | | <0.0001 |
| **SF12-PCS** |  | |  | |  | | |  |
| ISS 12+ | Not available | | 24.1 (7.3) | | 38.0 (11.8) | | | 41.0 (11.5) |
| 9-11 | Not available | | 26.0 (8.6) | | 42.1 (10.7) | | | 43.3 (12.3) |
| 4-8 | Not available | | 31.2 (9.8) | | 47.0 (10.6) | | | 49.0 (9.7) |
| 1-3 | Not available | | 38.6 (11.5) | | 47.6 (10.3) | | | 48.8 (10.2) |
| p value |  | | <0.0001 | | <0.0001 | | | <0.0001 |
| **SF12-MCS** |  | |  | |  | | |  |
| ISS 12+ | Not available | | 47.4 (11.1) | | 48.6 (14.0) | | | 49.9 (9.4) |
| 9-11 | Not available | | 47.3 (11.5) | | 50.1 (11.4) | | | 51.4 (12.3) |
| 4-8 | Not available | | 50.2 (11.7) | | 52.7 (10.2) | | | 53.7 (9.4) |
| 1-3 | Not available | | 48.2 (12.2) | | 51.5 (10.8) | | | 51.9 (10.3) |
| p value |  | | 0.001 | | 0.007 | | | 0.006 |
|  |  | |  | |  | | |  |
|  | | **Mean (SD)** | | **Mean (SD)** | | **Mean (SD)** | **Mean (SD)** | |
| **WHODAS** | |  | |  | |  |  | |

| ISS 12+ | Not available | Not available | 23.7 (24.4) | 20.4 (22.2) |
| --- | --- | --- | --- | --- |

| 9-11 | Not available | Not available | 17.0 (20.4) | 17.5 (20.1) |
| --- | --- | --- | --- | --- |
| 4-8 | Not available | Not available | 11.1 (16.9) | 8.7 (15.5) |
| 1-3 | Not available | Not available | 11.3 (17.7) | 10.5 (18.2) |
| p value |  |  | <0.0001 | <0.0001 |
| **IES-R total** |  |  |  |  |
| ISS 12+ | Not available | 3.77 (2.84) | 3.62 (3.26) | 3.26 (2.87) |
| 9-11 | Not available | 3.91 (3.05) | 2.83 (3.00) | 3.22 (3.49) |
| 4-8 | Not available | 3.34 (2.92) | 2.16 (2.81) | 1.77 (2.55) |
| 1-3 | Not available | 3.79 (3.27) | 2.42 (2.89) | 1.86 (2.70) |
| p value |  | 0.02 | 0.0009 | <0.0001 |
| **DASS-21 total** |  |  |  |  |
| ISS 12+ | Not available | 14.0 (14.8) | 14.9 (15.6) | 13.5 (13.2) |
| 9-11 | Not available | 13.4 (15.1) | 11.3 (14.9) | 12.3 (17.0) |
| 4-8 | Not available | 11.5 (13.9) | 9.2 (13.8) | 7.2 (11.8) |
| 1-3 | Not available | 13.4 (16.0) | 10.5 (15.0) | 8.4 (13.5) |
| p value |  | 0.049 | 0.019 | 0.0004 |
| **Perceived change / progress (-5 to +5)** |  |  |  |  |
| ISS 12+ |  |  | 1.69 (2.54) | 2.80 (1.87) |
| 9-11 |  |  | 2.66 (2.14) | 2.59 (2.34) |
| 4-8 |  |  | 2.87 (2.27) | 3.38 (2.12) |
| 1-3 |  |  | 3.10 (2.43) | 3.45 (2.15) |
| p value |  |  | <0.0001 | 0.0014 |
| **Numeric pain scale among those reporting any pain at each interview*** |  |  |  |  |
| ISS 12+ |  | 5.9 (2.1) | 4.0 (2.7) | 3.8 (2.2) |
| 9-11 |  | 5.5 (2.3) | 4.0 (2.2) | 4.0 (2.2) |
| 4-8 |  | 4.9 (2.2) | 3.3 (2.2) | 3.1 (2.2) |
| 1-3 |  | 4.7 (2.3) | 3.9 (2.4) | 3.7 (2.3) |
| p value |  | <0.0001 | 0.0006 | 0.007 |
| **Pain catastrophizing scale among those reporting any pain at each interview *** |  |  |  |  |
| ISS 12+ |  | 18.5 (15.2) | 17.6 (14.8) | 16.0 (13.6) |
| 9-11 |  | 18.1 (14.3) | 14.5 (13.3) | 15.8 (14.4) |
| 4-8 |  | 15.2 (13.4) | 12.6 (13.7) | 11.3 (13.5) |
| 1-3 |  | 16.0 (13.9) | 15.4 (14.4) | 14.5 (14.0) |
| p value |  | 0.056 | 0.014 | 0.012 |
| **OMPSQ among those reporting current pain at each interview** |  |  |  |  |
| ISS 12+ |  | 50.7 (16.0) | 45.0 (23.6) | 46.3 (21.0) |
| 9-11 |  | 48.4 (16.4) | 41.8 (20.1) | 43.1 (21.5) |
| 4-8 |  | 43.2 (16.5) | 37.1 (19.8) | 35.1 (20.4) |
| 1-3 |  | 40.6 (18.7) | 37.4 (21.2) | 36.5 (21.5) |
| p value |  | <0.0001 | 0.023 | 0.003 |
|  |  |  |  |  |

*26 individuals who did not report pain at baseline missed out on the questions about pain and pain catastrophizing at their 6 month follow up interview

Table A3 Adjusted mixed models for repeated measures over time

|  | **Mean difference  β (95% CI)** | **p value** | **Mean difference  β (95% CI)** | **p value** | **Interaction p value** |
| --- | --- | --- | --- | --- | --- |
|  | **PRE INJURY** |  | **12 MONTH  FOLLOW UP** |  |  |
| **PAID WORK** |  |  |  |  |  |
| **Model 1: Minimally sufficient adjustment at or before injury*** |  |  |  |  | 0.009 |
| ISS 12+ (severe) | -0.30 (-0.95, 0.34) | 0.35 | -1.29 (-2.02, -0.57) | 0.0004 |  |
| ISS 9-11 | 0.29 (-0.26, 0.83) | 0.30 | -0.40 (-0.99, 0.20) | 0.19 |  |
| ISS 4-8 | -0.13 (-0.39, 0.14) | 0.34 | -0.40 (-0.74, -0.07) | 0.015 |  |
| ISS 1-3 (minor) | Ref |  | Ref |  |  |
| **Model 2: Full adjustment including post-injury factors*** |  |  |  |  | 0.02 |
| ISS 12+ (severe) | -0.50 (-1.20, 0.18) | 0.14 | -1.46 (-2.22, -0.70) | 0.0001 |  |
| ISS 9-11 | 0.17 (-0.40, 0.73) | 0.6 | -0.49 (-1.10, 0.13) | 0.12 |  |
| ISS 4-8 | -0.17 (-0.46, 0.11) | 0.2 | -0.46 (-0.81, -0.11) | 0.01 |  |
| ISS 1-3 (minor) |  |  |  |  |  |
|  | **BASELINE  POST INJURY** |  | **12 MONTH  FOLLOW UP** |  |  |
| **SF12 PCS** |  |  |  |  |  |
| **Model 1: Minimally sufficient adjustment at or before injury*** |  |  |  |  | <0.0001 |
| ISS 12+ (severe) | -13.4 (-16.0, -10.9) | <0.0001 | -7.01 (-9.86, -4.17) | <0.0001 |  |
| ISS 9-11 | -11.4 (-13.4, -9.3) | <0.0001 | -3.88 (-6.01, -1.74) | 0.0004 |  |
| ISS 4-8 | -7.49 (-8.51, -6.47) | <0.0001 | 0.17 (-0.98, 1.31) | 0.7 |  |
| ISS 1-3 (minor) | Ref |  | Ref |  |  |
| **Model 2: Full adjustment including post-injury factors*** |  |  |  |  | <0.0001 |
| ISS 12+ (severe) | -8.73 (-11.0, -6.52) | <0.0001 | -2.31 (-4.98, 0.36) | 0.09 |  |
| ISS 9-11 | -8.21 (-9.91, -6.50) | <0.0001 | -0.84 (-3.84, 1.17) | 0.4 |  |
| ISS 4-8 | -5.93 (-6.81, -5.04) | <0.0001 | 1.71 (0.63, 2.79) | 0.002 |  |
| ISS 1-3 (minor) | Ref |  | Ref |  |  |
| **SF12 MCS** |  |  |  |  |  |
| **Model 1: Minimally sufficient adjustment at or before injury*** |  |  |  |  | 0.5 |
| ISS 12+ (severe) | -0.86 (-3.57, 1.84) | 0.53 | -2.29 (-5.20, 0.63) | 0.12 |  |
| ISS 9-11 | -0.97 (-3.09, 1.15) | 0.37 | 0.09 (-2.11, 2.29) | 0.94 |  |
| ISS 4-8 | 1.15 (0.05, 2.25) | 0.04 | 0.54 (-0.64, 1.73) | 0.37 |  |
| ISS 1-3 (minor) | Ref |  | Ref |  |  |
| **Model 2: Full adjustment including post-injury factors*** |  |  |  |  | 0.4 |
| ISS 12+ (severe) | 0.90 (-1.18, 2.99) | 0.4 | -0.63 (-3.23, 1.97) | 0.6 |  |
| ISS 9-11 | -0.14 (-1.74, 1.46) | 0.8 | 0.49 (-1.46, 2.44) | 0.6 |  |
| ISS 4-8 | 1.08 (0.25, 1.91) | 0.01 | 0.38 (-0.18, 1.43) | 0.5 |  |
| ISS 1-3 (minor) | Ref |  | Ref |  |  |
| **EQ5D TTO summary score** |  |  |  |  |  |
| **Model 1: Minimally sufficient adjustment at or before injury*** |  |  |  |  | <0.0001 |
| ISS 12+ (severe) | -0.32 (-0.41, -0.23) | <0.0001 | -0.18 (-0.25, -0.10) | <0.0001 |  |
| ISS 9-11 | -0.26 (-3.33, -0.19) | <0.0001 | -0.05 (-0.11,-0.001) | 0.047 |  |
| ISS 4-8 | -0.18 (-0.21, -0.14) | <0.0001 | -0.01 (-0.04, 0.03) | 0.6 |  |
| ISS 1-3 (minor) | Ref |  | Ref |  |  |
| **Model 2: Full adjustment including post-injury factors*** |  |  |  |  | <0.0001 |
| ISS 12+ (severe) | -0.19 (-0.27, -0.12) | <0.0001 | -0.06 (-0.13,-0.001) | 0.047 |  |
| ISS 9-11 | -0.17 (-0.23, -0.11) | <0.0001 | 0.008 (-0.04, 0.06) | 0.7 |  |
| ISS 4-8 | -0.14 (-0.17, -0.10) | <0.0001 | 0.02 (-0.005, 0.05) | 0.11 |  |
| ISS 1-3 (minor) | Ref |  | Ref |  |  |
| **WHODAS** |  |  |  |  |  |
| **Model 1: Minimally sufficient adjustment at or before injury*** |  |  |  |  |  |
| ISS 12+ (severe) | - |  | 9.05 (3.78, 14.3) | 0.0008 |  |
| ISS 9-11 | - |  | 5.34 (1.45, 9.24) | 0.007 |  |
| ISS 4-8 | - |  | -0.36 (-2.52, 1.79) | 0.7 |  |
| ISS 1-3 (minor) | - |  | Ref |  |  |
| **Model 2: Full adjustment including post-injury factors*** |  |  |  |  |  |
| ISS 12+ (severe) | - |  | 3.55 (-0.91, 8.02) | 0.12 |  |
| ISS 9-11 | - |  | 2.51 (-0.81, 5.84) | 0.14 |  |
| ISS 4-8 | - |  | -0.88 (-2.71, 0.95) | 0.3 |  |
| ISS 1-3 (minor) | - |  | Ref |  |  |

Footnotes:

*Interaction p value for interaction between time point and ISS category

**Minimally sufficient adjustment factors for both exposure and outcome (Model 1): age, sex, crash role, perceived danger in crash, preinjury health, preinjury EQ5D, recruitment source.

Full adjustment for all factors hypothesised to underlie either exposure or outcome status (Model 2), including post-injury factors: covariates from Model 1 PLUS education, preinjury work, social satisfaction, preinjury history of anxiety or depression, pain at baseline, pain catastrophising at baseline, DASS-21 and IESR scores at baseline, and CTP claimant status.
